# P-2068. Assessing New York City’s COVID-19 Vaccine Rollout Strategy: A Case for Risk-Informed Distribution

**DOI:** 10.1093/ofid/ofae631.2224

**Published:** 2025-01-29

**Authors:** Nina Schwalbe, Marta C Nunes, Clare Cutland, Brian Wahl, Daniel Reidpath

**Affiliations:** University of the Witwatersrand, New York, New York; Université Claude Bernard Lyon 1, Lyon, Auvergne, France; University of the Witwatersrand, New York, New York; Johns Hopkins Bloomberg School of Public Health, Baltimore, Maryland; Queen Margaret University, Edinburgh, Scotland, United Kingdom

## Abstract

**Background:**

This study reviews the impact of eligibility policies in the early rollout of the COVID-19 vaccine on coverage and probable outcomes, with a focus on New York City (NYC).

**Methods:**

A retrospective ecological study was conducted assessing age 65+, area-level income, vaccination coverage, and COVID-19 mortality rates, using linked Census Bureau data and NYC Health administrative data aggregated at the level of modified zip code tabulation areas (MODZCTA). The population for this study was all individuals in 177 MODZCTA in NYC. Population data were obtained from Census Bureau and NYC Health administrative data. The total mortality rate was examined through an ordinary least squares (OLS) regression model, using area-level wealth, the proportion of the population aged 65 and above, and the vaccination rate among this age group as predictors.

**Results:**

Low-income areas with high proportions of older people demonstrated lower coverage rates (mean vaccination rate 52.8%; maximum coverage 67.9%) than wealthier areas (mean vaccination rate 74.6%; maximum coverage 99% in the wealthiest quintile) in the first 3 months of vaccine rollout and higher mortality over the year. Despite vaccine shortages, many younger people accessed vaccines ahead of schedule, particularly in high-income areas (mean coverage rate 60% among those 45–64 years in the wealthiest quintile).

**Conclusion:**

A vaccine program that prioritized those at greatest risk of COVID-19-associated morbidity and mortality would have prevented more deaths than the strategy that was implemented. When rolling out a new vaccine, policymakers must account for local contexts and conditions of high-risk population groups. If New York had focused limited vaccine supply on low-income areas with high proportions of residents 65 or older, overall mortality might have been lower.

**Disclosures:**

All Authors: No reported disclosures

